# Chemically Assisted Thermo-Mechanical Devulcanization of Passenger Car Tire Rubber for SBS-Composite-Modified Asphalt: Mechanisms, Rheological Properties, and Dosage Effects

**DOI:** 10.3390/polym18172102

**Published:** 2026-08-29

**Authors:** Bo Zhang, Shuai Zhang, Danjun Duan, Wenwen Yu, Jiayu Bi, Wei Wang, Yachun Wei, Runtian Chang

**Affiliations:** 1 National & Local Joint Engineering Laboratory of Advanced Road Materials, Shanxi Jiaoke New Materials Science and Technology Co., Ltd., Taiyuan 030032, China; 2 Shanxi Intelligent Transportation Laboratory Co., Ltd., Taiyuan 030032, China; 3 Shanxi Transportation Technology Research and Development Co., Ltd., Taiyuan 030032, China; 4 College of Materials Science & Engineering, Taiyuan University of Technology, Taiyuan 030024, China

**Keywords:** passenger car waste tire, crumb rubber, devulcanization, SBS composite modification, modified asphalt, rheological properties

## Abstract

Passenger car waste tires (PCWTs) are difficult to recycle as asphalt modifiers due to their high styrene-butadiene rubber (SBR) content and poor compatibility with asphalt. This study investigates whether hexadecylamine (HDA)-assisted thermo-mechanical devulcanization can transform PCWT rubber into an effective modifier for styrene-butadiene-styrene (SBS)-composite-modified asphalt, and how rubber dosage influences the performance balance. PCWT rubber was devulcanized using HDA-assisted twin-screw extrusion and incorporated into 90# asphalt with 3 wt% SBS at 20 wt% and 30 wt% dosages, alongside non-devulcanized PCWT and truck tire rubber for comparison. Comprehensive characterization, including sol fraction, Fourier-transform infrared spectroscopy (FTIR), scanning electron microscopy/energy-dispersive X-ray spectroscopy (SEM/EDS), dynamic shear rheometer (DSR), multiple stress creep recovery (MSCR), and bending beam rheometer (BBR), was conducted. The results demonstrate that HDA-assisted devulcanization effectively breaks sulfur crosslinks while preserving the rubber backbone, significantly improving compatibility and low-temperature performance. Increasing the devulcanized rubber dosage from 20 wt% to 30 wt% compensates for the reduced high-temperature stiffness, achieving a balanced performance profile comparable to truck tire rubber. The novelty of this work lies in establishing HDA-assisted thermo-mechanical devulcanization as a viable pathway for converting otherwise underutilized PCWT rubber into a high-performance asphalt modifier, thereby expanding the feedstock options for crumb rubber-modified asphalt.

## 1. Introduction

Note on terminology: In this manuscript, the term asphalt binder (or bitumen) refers to the pure petroleum-derived binding material, while asphalt mixture refers to the composite of asphalt binder with mineral aggregates. All experiments in this study were conducted on asphalt binders, not asphalt mixtures.

The rapid growth of the automobile industry leads to an annual output of more than 17 million tons of waste tires worldwide. In China alone, about 13 million tons of waste tires are produced every year, with an annual growth rate of 8–10%. Among these, passenger car waste tires (PCWTs) account for a significant proportion, yet their recycling rate remains below 50% due to technical limitations [1,2]. Unregulated incineration and landfilling, which are the main non-compliant disposal methods in areas with imperfect recycling systems, will cause serious environmental pollution and a huge waste of high-value rubber resources. The current mainstream industrial standardized recycling paths for waste tires mainly include pyrolysis to recover oil and carbon black, and mechanochemical regeneration to produce reclaimed rubber, which have been widely applied in engineering practice [3,4,5].

Bitumen additives are generally classified into several categories: polymer modifiers (thermoplastics, elastomers, and thermoplastic elastomers), plasticizers, adhesion promoters, anti-aging agents, fillers, and secondary raw materials [6]. Among these, polymer modification—particularly with thermoplastic elastomers such as SBS—has been most widely adopted for improving high- and low-temperature performance. Crumb rubber from waste tires falls into the category of secondary raw materials used as polymer modifiers [7].

Crumb rubber-modified asphalt (CRMA) has emerged as one of the most promising solutions for waste tire valorization. Extensive studies have demonstrated that incorporating crumb rubber into asphalt binders significantly enhances high-temperature rutting resistance, low-temperature cracking resistance, fatigue performance, and noise reduction [8,9,10]. However, the rubber composition differs markedly between tire types. Passenger car tires are predominantly manufactured from styrene-butadiene rubber (SBR, ~65%) blended with natural rubber (NR, ~35%), whereas truck tires contain a much higher proportion of NR (~65%) with a smaller fraction of SBR (~35%) [11]. The glass transition temperature of SBR is approximately 20 °C higher than that of NR, endowing PCWTs with superior thermal stability but also rendering them more difficult to devulcanize and compatibilize with asphalt binders [12,13,14]. Consequently, nearly all existing CRMA research and field applications have focused on truck tire rubber (TR), while PCWT rubber (PCWTR) has been largely overlooked.

Crumb rubber-modified asphalt is one of the most promising ways to realize the high-value utilization of waste tires in road engineering. A large number of engineering practices have proved that adding crumb rubber to asphalt binder can significantly improve the high-temperature rutting resistance, low-temperature crack resistance, fatigue performance and noise reduction performance of asphalt pavement, with outstanding technical and economic benefits. Compared with other devulcanization technologies, microwave devulcanization has the defect of uneven energy distribution and unstable product quality, while biological devulcanization has the problem of excessively long treatment cycle and cannot realize large-scale continuous industrial production [15]. The HDA-assisted thermo-mechanical devulcanization selected in this study can achieve efficient selective cleavage of sulfur crosslinks while retaining the rubber backbone, which has significant engineering application advantages [16,17].

Various physical and chemical modification strategies have been explored to overcome the poor compatibility of PCWTR with asphalt [7,18,19]. Among these, twin-screw extrusion devulcanization—a thermo-mechanical process—has emerged as a promising technology for breaking the crosslinking network [20,21,22]. Recently, chemical-assisted devulcanization using reagents such as hexadecylamine (HDA) has been shown to further enhance the efficiency of crosslink cleavage by promoting nucleophilic attack on sulfur atoms [17,23,24,25]. Among amine-based devulcanizing agents, hexadecylamine (HDA) was selected for this study based on several considerations. First, as a long-chain primary amine (C_16_H_35_NH_2_), HDA exhibits good thermal stability at the typical extrusion temperatures (140–180 °C) used in this work. Second, comparative studies have shown that amines, particularly HDA, demonstrate the largest decrease in remaining crosslink density with increasing concentration compared to other devulcanizing agents [26]. Third, HDA is commercially available at reasonable cost and has been successfully applied in the devulcanization of various diene rubbers [27]. Alternative amine-based reagents, such as shorter-chain amines or secondary/tertiary amines, were not selected due to their lower thermal stability or reduced nucleophilicity toward sulfur crosslinks. This combined chemical and thermo-mechanical approach can effectively break C–S and S–S bonds while preserving the polymer backbone, thereby improving the compatibility of PCWTR with asphalt [28].

Another effective approach to enhancing CRMA performance is the incorporation of styrene-butadiene-styrene (SBS), a widely used thermoplastic elastomer [29,30,31]. Composite modification using both rubber powder and SBS can produce synergistic effects, improving the overall mechanical and rheological properties of asphalt [32]. The SBS phase forms a three-dimensional network that provides high elastic recovery, while the rubber particles act as a dispersed reinforcing phase [33,34]. This combination can achieve performance comparable to SBS-modified asphalt but with lower SBS content, thereby reducing material costs [35,36].

Despite these advances, systematic evaluations of PCWTR as an asphalt modifier remain scarce. In particular, two critical gaps persist in the literature: (1) the effectiveness of chemical-assisted thermo-mechanical devulcanization specifically for PCWTR, and (2) the performance of PCWTR/SBS composite-modified asphalt at both conventional (20 wt%) and high (30 wt%) dosages. High-dosage CRMA has attracted increasing attention due to its potential to consume larger volumes of waste tires and produce highly elastic pavements with superior crack resistance [37,38]. However, the compatibility challenges associated with high rubber dosages are exacerbated for PCWTR due to its high SBR content.

Therefore, this study aims to address these knowledge gaps by systematically evaluating the performance of SBS-composite-modified asphalt binders incorporating three crumb rubber types—non-devulcanized PCWTR (PC-raw), HDA-assisted thermo-mechanically devulcanized PCWTR (PC-dev), and non-devulcanized truck tire rubber (TR-raw)—each blended with 3 wt% SBS at two rubber dosages (20 wt% and 30 wt% by weight of asphalt). The research objectives are: (1) to characterize the chemical and structural changes induced by HDA-assisted devulcanization of PCWTR; (2) to evaluate and compare the physical, rheological, and low-temperature properties of the six resulting modified asphalts; and (3) to elucidate the dosage-dependent performance mechanisms. The findings are expected to provide technical guidance for the high-value utilization of passenger car waste tires in asphalt pavement applications.

## 2. Materials and Methods

**Figure 1:**
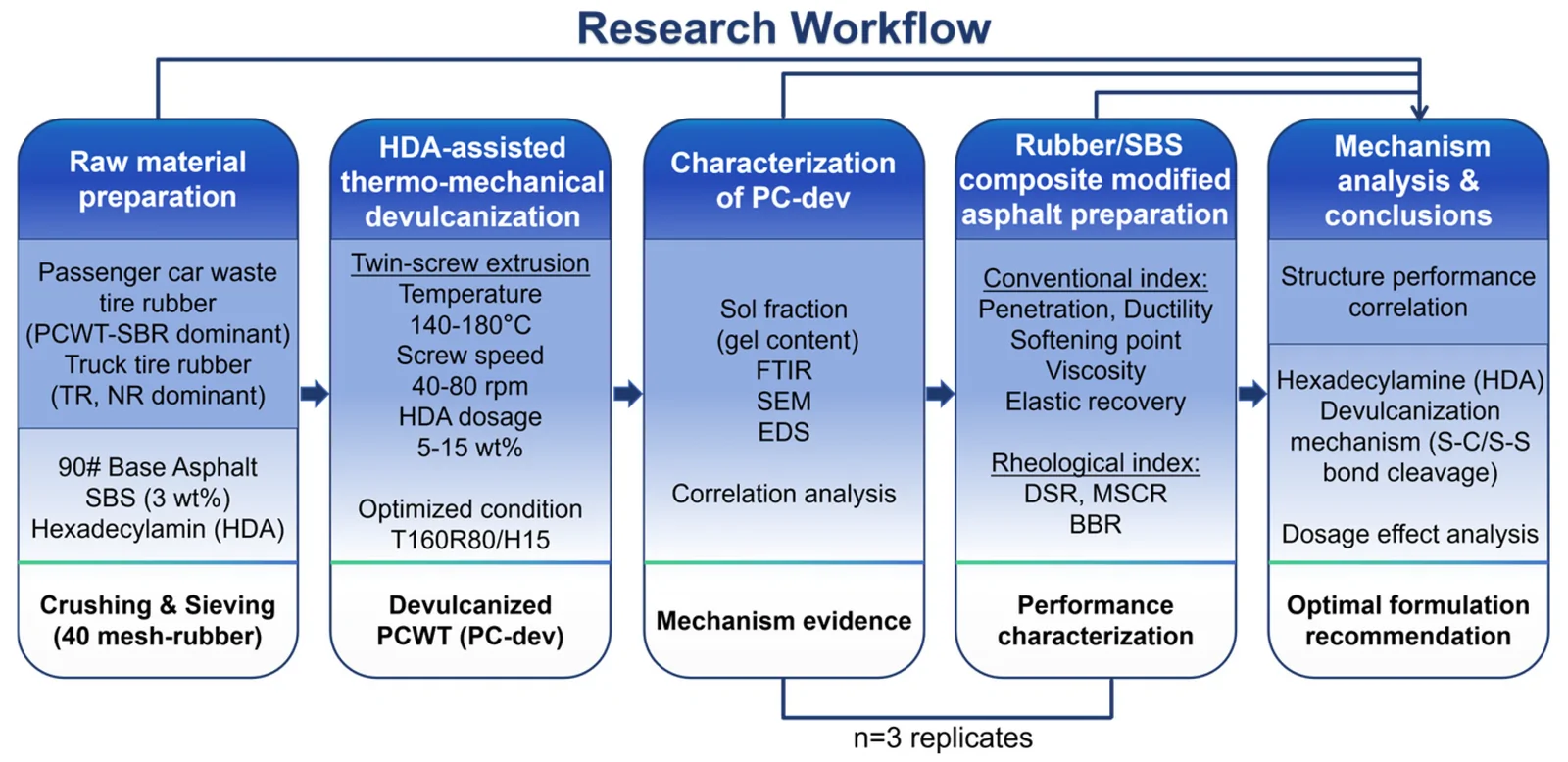
Research flowchart.

This section describes the materials used, the HDA-assisted thermo-mechanical devulcanization process, the preparation of rubber/SBS composite-modified asphalt, and the comprehensive characterization methods employed in this study. The overall experimental framework is illustrated in Figure 1.

### 2.1. Materials

The base asphalt (Kunlun 90#) was purchased from Sinopec Group, with basic properties listed in Table 1. The SBS modifier was also obtained from Sinopec Group, with technical specifications in Table 2.

The truck radial tire rubber powder (30 mesh, 600 μm) was purchased from Hubei Anjie Road and Bridge Technology Co., Ltd. (Hubei, China) The passenger car tire powder (30 mesh, 600 μm) was purchased from Huzhou Pulaijin Technology Co., Ltd. (Huzhou, China) Hexadecylamine (HDA, C_16_H_35_NH_2_, ≥98% purity) was purchased from Sigma-Aldrich (St. Louis, MO, USA).

### 2.2. Rubber Devulcanization

The devulcanized passenger car tire rubber powder (PC-dev) was prepared via a chemical-assisted thermo-mechanical devulcanization process, in which hexadecylamine (HDA, C_16_H_35_NH_2_, ≥98% purity) served as the devulcanizing agent and engine oil functioned as both a dispersing medium and a swelling promoter to facilitate the penetration of HDA into the rubber network.

Raw 30-mesh (600 μm) passenger car tire rubber powder was mixed with engine oil at a mass ratio of 20:3. HDA was then added at dosages of 5, 10, or 15 wt% relative to the rubber powder mass. The mixture was mechanically stirred at 300 rpm for 5 min to ensure uniform coating and partial penetration of the reagents. The resulting compound was fed into a co-rotating twin-screw extruder (Process 11, Thermo Fisher Scientific, Waltham, MA, USA; screw diameter: 16 mm; L/D ratio: 20:1) for high-temperature shearing. The barrel temperature was set to 140, 160, or 180 °C, and the screw rotational speed was adjusted to 40, 60, or 80 rpm. The shear torque was maintained in the range of 70–100 N·m throughout extrusion.

Seven PC-dev samples were prepared under different combinations of processing parameters, as summarized in Table 3. The sample nomenclature follows the format PC-dev-TXRYHZ, where T, R, and H denote the barrel temperature (°C), screw rotational speed (rpm), and HDA content (wt%), respectively.

After extrusion, the devulcanized rubber strands were cooled in a water bath (25 °C), pelletized, and ground to pass an 80-mesh (180 μm) sieve. The resulting PC-dev powders were dried at 60 °C for 12 h to remove residual moisture and volatiles prior to use.

### 2.3. Preparation of Rubber-Modified Asphalt

First, the base asphalt was placed in an oven and kept at 110 °C for 2 h to bring it to a flowing state. Then, the base asphalt was weighed and placed in a heater to be heated to 150 °C. Subsequently, different types of rubber powder and modifiers were quantitatively added to the asphalt, and the mixture was stirred at a speed of 1000 rpm using an electric stirrer for 1 h. Then, the modified asphalt preparation process used a tooth-type high-shear emulsifier (FM300, FLUKO, Shanghai, China) equipped with a 60 mm diameter stirring head, and the total volume of the adopted mixing vessel was 1 L. The blend was sheared at 5000 rpm for 120 min at 190 °C. Under this set of process conditions, the calculated shear Reynolds number of the asphalt binder system reached 1.2 × 10^5^, which belongs to a typical strong turbulent mixing state and can ensure that rubber particles and SBS modifiers are uniformly dispersed in the asphalt matrix without agglomeration. The proportions mentioned in this article are all external additions. Samples were named PC-raw-20, PC-raw-30, PC-dev-20, PC-dev-30, TR-raw-20 and TR-raw-30. The component contents are shown in Table 4.

### 2.4. Characterization

#### 2.4.1. Sol Fraction Determination

The sol fraction of the crumb rubber was measured to evaluate the degree of devulcanization. The rubber powder was first dried in a vacuum oven at 60 °C for 6 h and weighed (recorded as *m*_1_). The dried sample was wrapped in a wire mesh and placed in a Soxhlet extractor. The rubber was extracted with acetone for 12 h, followed by toluene for 24 h. After extraction, the residue was dried in a vacuum oven at 60 °C for 8 h and weighed (recorded as *m*_2_). The sol fraction was calculated using the following equation:

(1) $$\mathrm{Sol}\;\mathrm{fraction}\;(\%)\;=\;\frac{m_{1}\;-\;m_{2}}{m_{1}}$$

where *m*_1_ is the mass of the rubber powder before extraction, and *m*_2_ is the mass of the insoluble residue after sequential acetone and toluene extraction.

#### 2.4.2. Fourier Transform Infrared (FTIR) Spectroscopy

This paper used a Fourier infrared spectrometer (FT-IR, Tensor 27, Bruker Optics, Ettlingen, Germany) equipped with an attenuated total reflection (ATR) accessory to study the molecular structures of different rubber powders. The tests were conducted within the wavenumber range of 400 to 4000 cm^−1^ with a spectral resolution of 4 cm^−1^.

#### 2.4.3. Scanning Electron Microscopy (SEM)

The surface morphology of the crumb rubber powders was observed using a cold-field emission scanning electron microscope (SEM, S-4800, Hitachi High-Technologies, Tokyo, Japan). The rubber powder was evenly dispersed on the conductive adhesive surface of a sample stub and sputter-coated with gold. The observation was performed under high vacuum at an accelerating voltage of 15 kV. The elemental composition of the rubber powders was analyzed using energy dispersive spectroscopy (EDS) attached to the SEM.

#### 2.4.4. Physical Properties

The conventional physical properties of the single crumb rubber-modified asphalts were tested according to the standard JTG 3410-2025. The following properties were measured: penetration at 25 °C, softening point (ring-and-ball method), ductility at 5 °C, rotational viscosity at 180 °C, and elastic recovery at 25 °C.

#### 2.4.5. Dynamic Shear Rheometer (DSR) Test

The high-temperature performance of the crumb rubber-modified asphalts with different devulcanization degrees was evaluated using a dynamic shear rheometer (DSR, MCR 102e, Anton Paar, Graz, Austria). The multiple stress creep recovery (MSCR) test was performed according to AASHTO T315 and AASHTO MP 19-10. A 25 mm parallel plate geometry with a 1 mm gap was used. The test temperatures were 58 °C, 64 °C, 70 °C, and 76 °C.

#### 2.4.6. Multiple Stress Creep Recovery (MSCR) Tests

The MSCR (MCR 102e, Anton Paar, Austria) test procedure consisted of 20 creep-recovery cycles at a stress level of 0.1 kPa, followed immediately by 10 cycles at 3.2 kPa. Each cycle included a 1 s creep phase and a 9 s recovery phase. The total test duration was 300 s.

For each cycle, the peak strain at the end of the 1 s creep period (*ε_p_*) and the unrecovered strain at the end of the 10 s recovery period (*ε_u_*) were recorded. The percent recovery (*R*) and the non-recoverable creep compliance (*J_nr_*) were calculated using the following equations:

(2) $$R\;=\;\frac{\varepsilon_{p}\;-\;\varepsilon_{u}}{\varepsilon_{p}}\;\times \;100\%$$

(3) $$J_{nr}=\frac{\varepsilon_{u}}{\sigma }$$

where *σ* is the applied stress level (0.1 kPa or 3.2 kPa).

#### 2.4.7. Bending Beam Rheometer (BBR) Test

The low-temperature performance of the crumb rubber-modified asphalts was evaluated using a bending beam rheometer (BBR, TE-BBR, Cannon Instrument Company, State College, PA, USA) according to AASHTO T313. The BBR test measures the creep stiffness (S) and creep rate (m-value) under a constant load of 980 mN. Tests were performed at −18 °C and −24 °C. The deflection of the asphalt beam as a function of time was recorded, and the S-value and m-value were calculated according to the standard procedure.

### 2.5. Statistical Analysis

All experiments were performed in triplicate using three independently prepared samples. Results are reported as mean ± standard deviation (SD). Error bars in figures represent ±1 SD. Statistical significance was evaluated using one-way ANOVA followed by Tukey’s post hoc test (*p* < 0.05) where applicable.

## 3. Results

### 3.1. Sol Fraction Analysis

**Figure 2:**
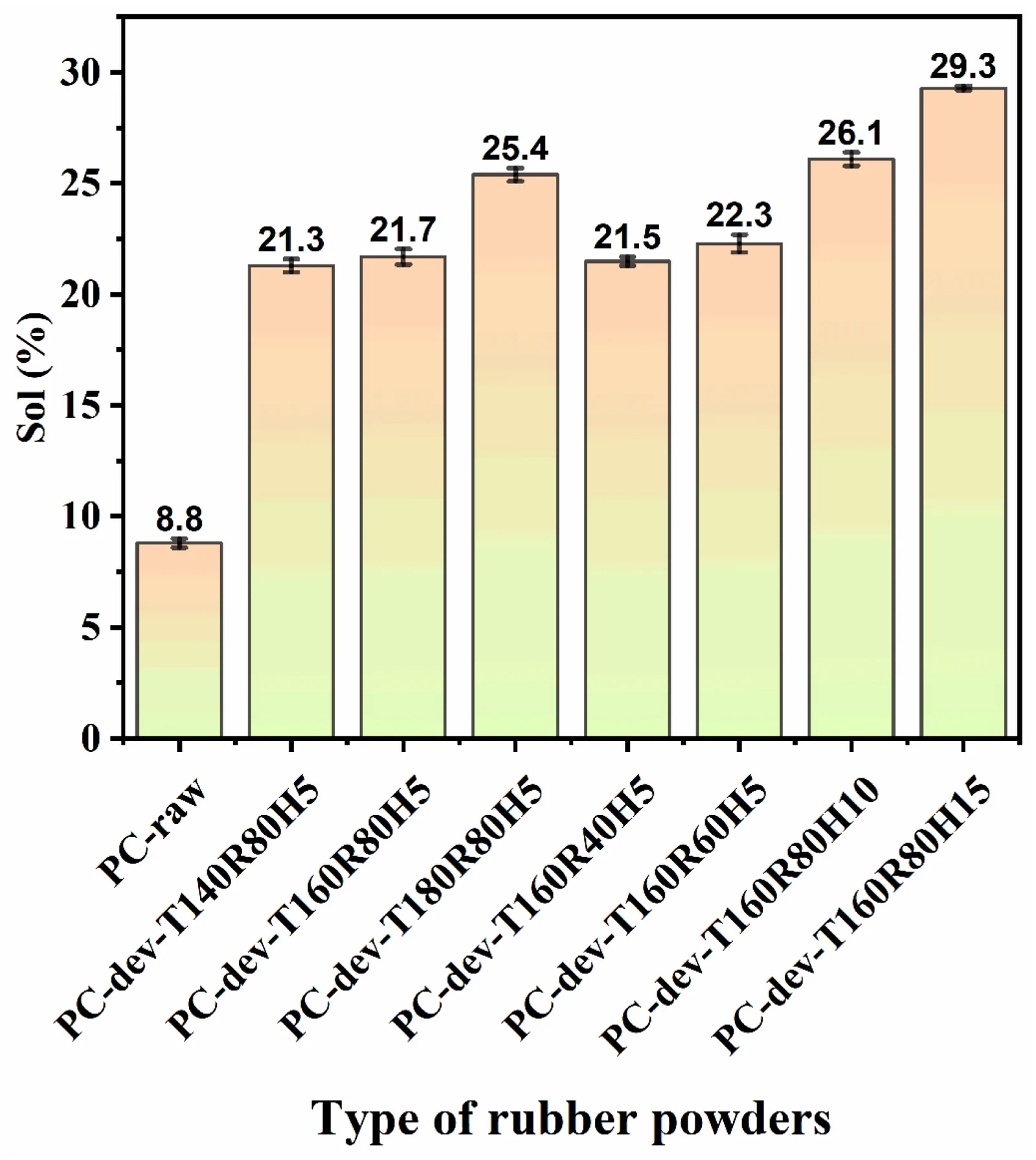
Sol fraction of PC-raw and PC-dev samples prepared under various devulcanization conditions.

Sol fraction serves as a quantitative indicator of the degree of devulcanization, reflecting the proportion of soluble polymer chains released from the crosslinked network [39]. As shown in Figure 2, PC-raw exhibits a sol fraction of only 8.8%, typical of untreated vulcanized rubber with an intact crosslinked structure. Among the seven PC-dev samples prepared under different conditions, the sol fraction increases progressively with HDA content and processing temperature, reaching a maximum of 29.3% for PC-dev-T160R80H15—a more than threefold increase compared to PC-raw. This significant increase confirms that the HDA-assisted thermo-mechanical treatment effectively breaks the sulfur crosslinking network. The highest sol fraction achieved (29.3%) is consistent with values reported in the literature for chemically assisted devulcanization. Temperature plays a critical role: increasing the barrel temperature from 140 °C to 180 °C raises the sol fraction from 21.3% to 25.4%, attributed to enhanced thermal activation and improved HDA penetration into the rubber matrix. HDA content exerts the most pronounced influence: raising HDA from 5 wt% to 15 wt% increases the sol fraction from 21.7% to 29.3%, demonstrating a clear dose–response relationship that confirms HDA’s active role as a nucleophilic devulcanizing agent. Based on these results, PC-dev-T160R80H15 (hereafter referred to as PC-dev) was selected for subsequent asphalt modification.

Based on these results, PC-dev-T160R80H15 (hereafter referred to as PC-dev) was selected for subsequent asphalt modification. The selection was based on three criteria: (1) it exhibited the highest sol fraction (29.3%), indicating the greatest extent of crosslink cleavage; (2) FTIR analysis showed no significant change in the C–H stretching region (2911/2843 cm^−1^), confirming that the rubber backbone remained intact without excessive main-chain degradation; and (3) the material remained processable after extrusion, with no signs of excessive stickiness, charring, or thermal degradation. These combined criteria ensure that the selected PC-dev represents an optimal balance between devulcanization efficiency and material integrity. While HDA represents a cost factor for industrial-scale application, the 29.3% sol fraction achieved at this dosage—more than three times that of untreated PC-raw (8.8%)—is essential for enabling PCWT rubber to achieve performance comparable to truck tire rubber. Future studies may explore intermediate HDA dosages or alternative processing conditions to optimize the cost-performance balance.

The sol fraction provides the most direct quantitative evidence of crosslink cleavage, as the release of soluble polymer chains from the vulcanized network can only occur when C–S and S–S bonds are broken [40]. The complementary techniques discussed in the following sections—FTIR, EDS, and SEM—provide supporting evidence at different structural levels.

It should be noted that during the twin-screw extrusion process, some volatilization of low-molecular-weight components may occur. The mass loss during extrusion was typically less than 2% of the total feed weight, attributed to evaporation of moisture and low-boiling-point oil fractions. A mild amine/rubber odor was noted during processing, indicating that adequate ventilation or gas purification would be necessary in an industrial-scale operation. The engine oil content in the rubber after extrusion was not quantitatively determined; however, visual inspection and FTIR analysis confirmed that the majority of the oil remained incorporated within the rubber matrix, as evidenced by the strong C–H absorption bands at 2911 and 2843 cm^−1^.

### 3.2. FTIR Analysis of Crumb Rubber Powders

**Figure 3:**
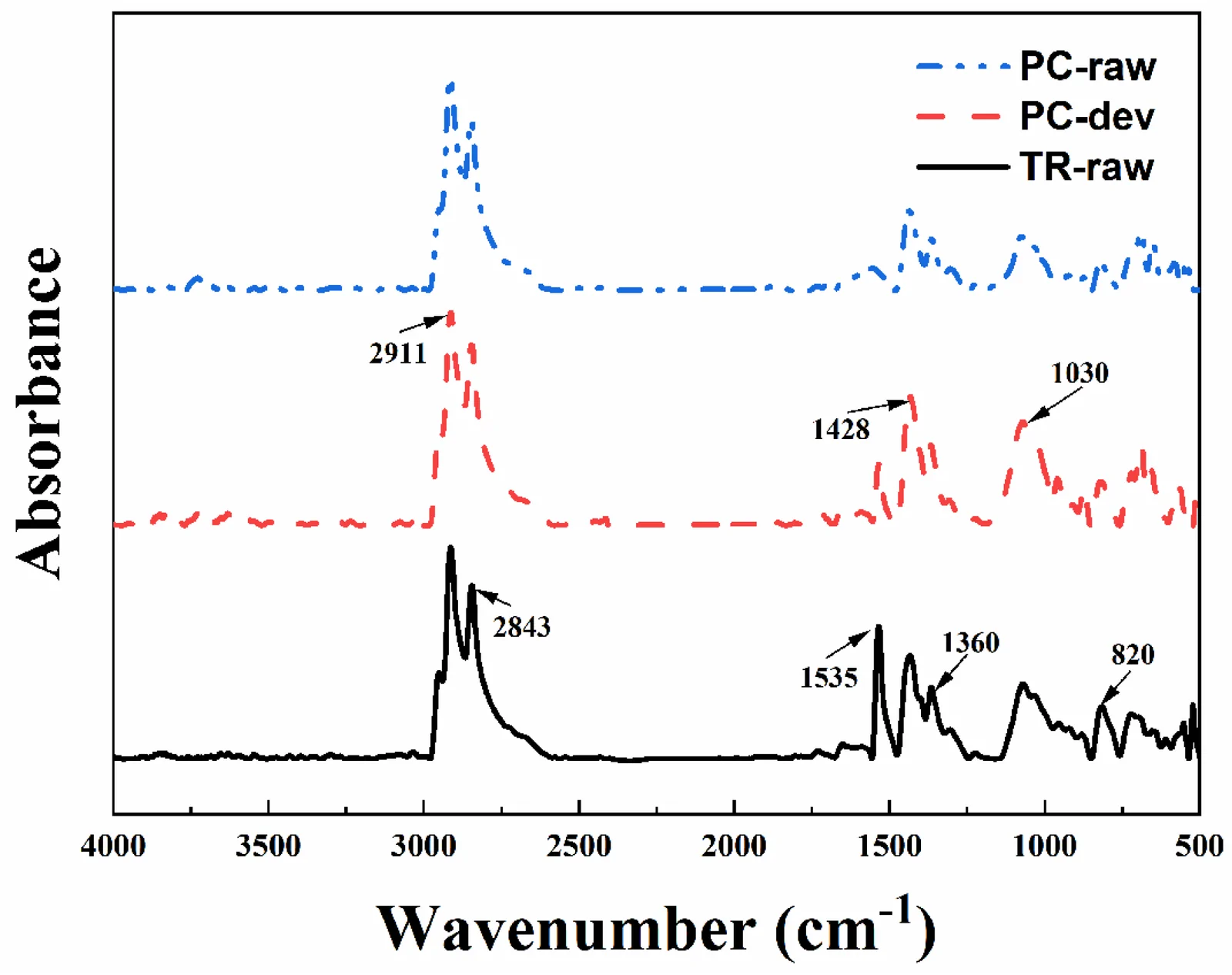
FTIR spectra of PC-raw, PC-dev, and TR-raw.

The ATR-FTIR spectra of PC-raw, PC-dev, and TR-raw are presented in Figure 3. All three samples exhibit characteristic absorption bands of hydrocarbon rubbers, including C–H stretching vibrations at 2911 and 2843 cm^−1^, CH_2_ bending at 1428 cm^−1^, and CH_3_ bending at 1360 cm^−1^. The band at 1535 cm^−1^ is attributed to C=C stretching, while the band at 820 cm^−1^ corresponds to aromatic C–H out-of-plane bending from styrene units. The intensity of the 820 cm^−1^ band is higher in PC-raw and PC-dev compared to TR-raw, consistent with the higher styrene-butadiene rubber (SBR) content (~65%) in passenger car tires versus the higher natural rubber (NR) content (~65%) in truck tires.

The most significant spectral difference between PC-dev and PC-raw is the markedly enhanced band at 1030 cm^−1^ in PC-dev, assigned to S=O stretching of sulfoxide groups. This provides direct spectroscopic evidence of C–S and S–S crosslink cleavage during devulcanization. Additionally, PC-dev shows an intensified band at 1710 cm^−1^ (C=O stretching), indicating thermo-oxidative modification accompanying the devulcanization process. The appearance of a weak band at approximately 3300 cm^−1^ (N–H stretching) in PC-dev suggests residual HDA or its reaction products with the rubber network. Importantly, no significant changes are observed in the C–H stretching region (2911/2843 cm^−1^), indicating that the rubber hydrocarbon backbone remains largely intact—the devulcanization process preferentially targets sulfur crosslinks rather than causing main-chain scission. These FTIR findings are fully consistent with the sol fraction results: the increased soluble fraction in PC-dev corresponds to the cleavage of crosslinks evidenced by the enhanced sulfoxide band.

It should be noted that the enhanced S=O band at 1030 cm^−1^ indicates oxidation of sulfur-containing species but does not, by itself, demonstrate C–S or S–S bond cleavage. However, in sulfur-vulcanized tire rubber, sulfur exists primarily in crosslinks rather than as free sulfur [20]. The appearance of sulfoxide species during devulcanization is widely interpreted as evidence that crosslinks have been broken and subsequently oxidized [41]. The simultaneous preservation of the C–H stretching region confirms that the cleavage is selective toward crosslinks rather than the polymer backbone. Thus, the 1030 cm^−1^ band serves as strong supporting evidence of crosslink cleavage when interpreted together with the sol fraction increase.

### 3.3. Scanning Electron Microscopy (SEM) Analysis

**Figure 4:**
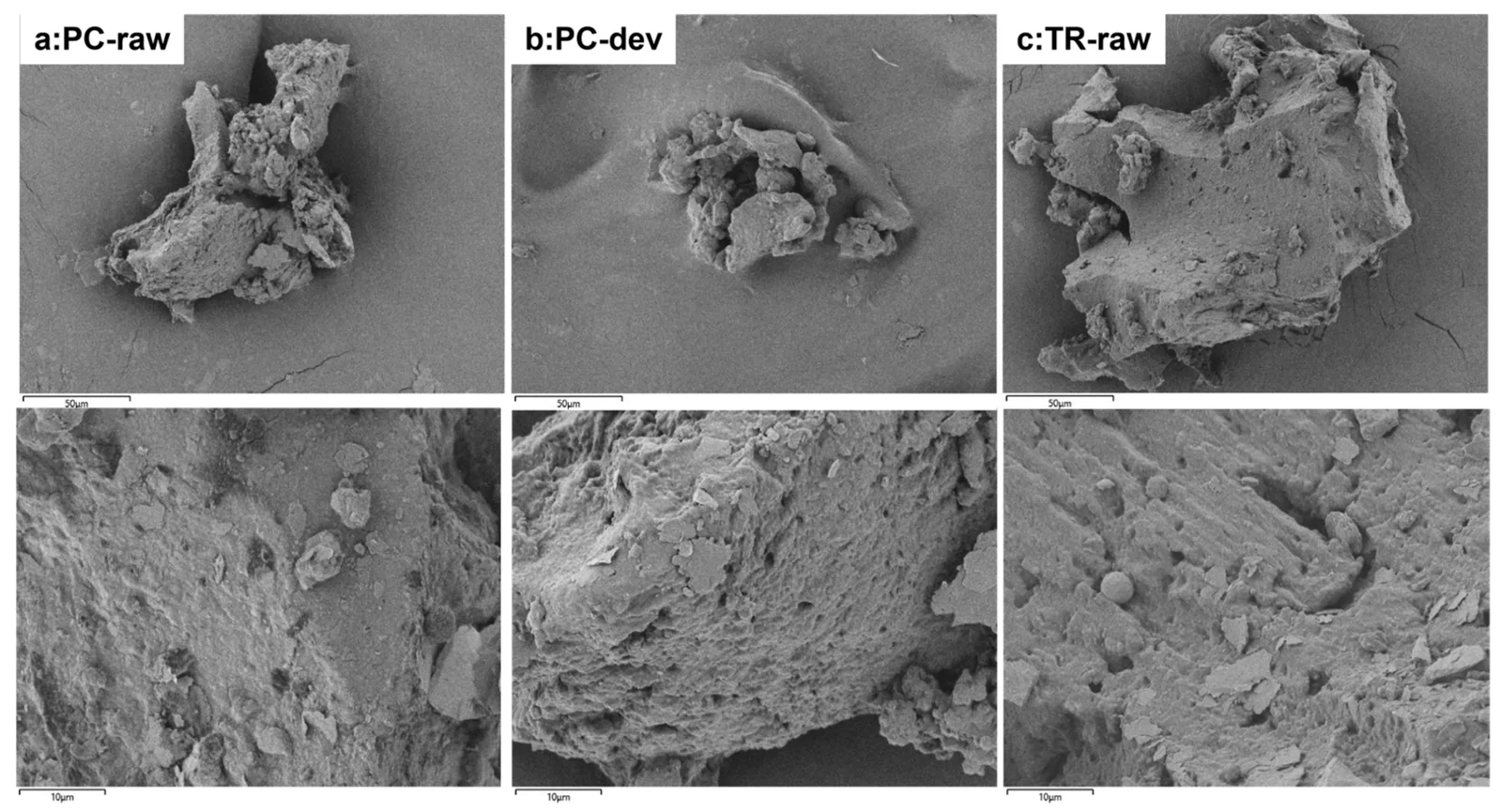
SEM micrographs of crumb rubber powders: PC-raw, PC-dev, and TR-raw (magnification: ×500; scale bar: 50 μm).

The SEM micrographs of PC-raw, PC-dev, and TR-raw are presented in Figure 4. PC-raw exhibits a relatively smooth and compact surface with limited porosity, characteristic of untreated vulcanized rubber with an intact crosslinked network. In contrast, PC-dev shows a markedly rougher, looser, and highly porous morphology with visible cracks and fragmented features. This structural transformation results from the HDA-assisted thermo-mechanical devulcanization, which breaks sulfur crosslinks and disrupts the rubber network, increasing the specific surface area [42]. TR-raw displays a moderately rough surface, smoother than PC-dev but more irregular than PC-raw, reflecting its different compounding and higher natural rubber content.

The rough, porous morphology of PC-dev is expected to enhance physical interlocking and interfacial adhesion with the asphalt matrix, while the increased sol fraction (29.3%) and FTIR-confirmed crosslink cleavage improve chemical compatibility and swelling behavior. These morphological changes provide direct evidence of successful devulcanization and support the improved performance of PC-dev-modified asphalt.

**Figure 5:**
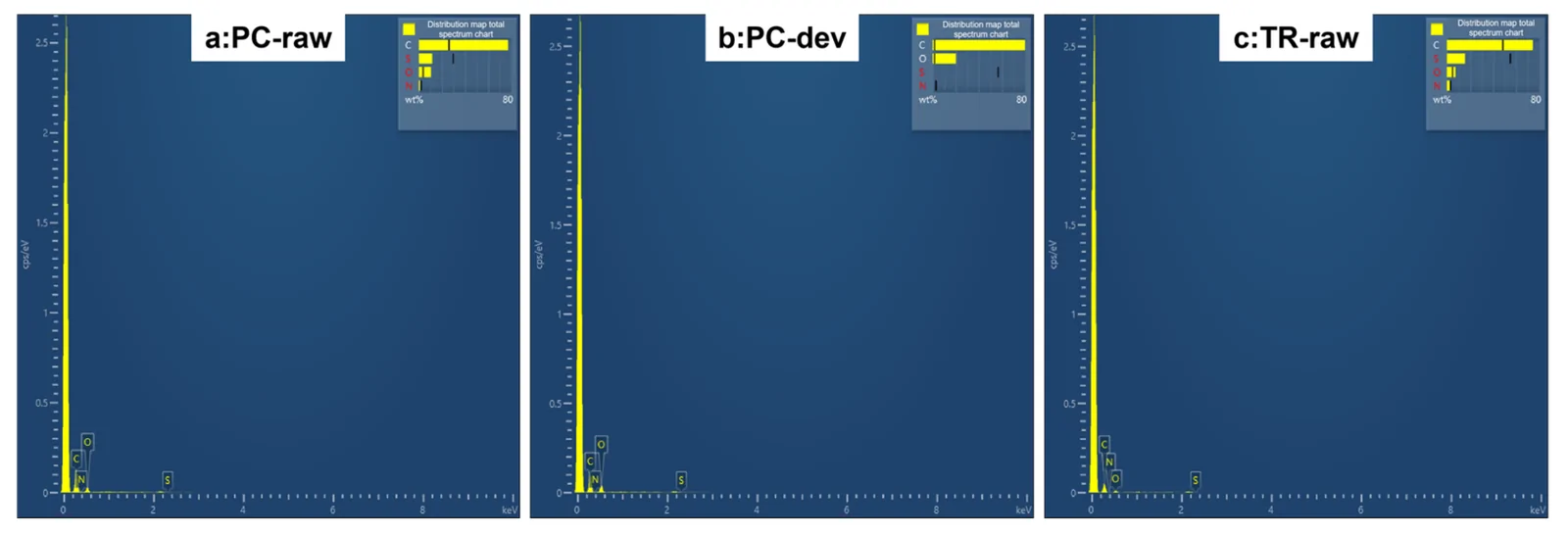
EDS spectrum of crumb rubber powders: PC-raw, PC-dev, and TR-raw.

EDS analysis was performed to complement the FTIR and SEM findings. As shown in Figure 5 and Table 5, the sulfur content of PC-dev drops dramatically from 11.4 wt% (PC-raw) to 0.6 wt%, while oxygen increases from 10.3 wt% to 20.1 wt%. This significant sulfur reduction provides direct elemental evidence of crosslink cleavage. The concurrent oxygen enrichment is consistent with the FTIR-observed intensification of S=O (1030 cm^−1^) and C=O (1710 cm^−1^) bands, confirming oxidative transformation of the cleaved sulfur species. The higher sulfur content in TR-raw (15.5 wt%) reflects the greater vulcanization degree typical of truck tire rubber. The combination of low sulfur content and high oxygen content in PC-dev, together with the rough porous morphology observed by SEM and the highest sol fraction (29.3%), confirms that the HDA-assisted thermo-mechanical treatment effectively breaks the sulfur crosslinking network of PCWTR without severely degrading the polymer backbone.

The EDS sulfur reduction from 11.4 to 0.6 wt% (a 95% decrease) is substantial; however, EDS alone does not distinguish crosslink sulfur from other sulfur-containing additives (e.g., vulcanization accelerators, anti-aging agents). In tire rubber, the vast majority of sulfur is incorporated into crosslinks [43]. The magnitude of the reduction observed here, combined with the sol fraction increase (direct evidence of crosslink cleavage) and the FTIR sulfoxide formation (indicating oxidation of cleaved crosslinks), provides strong corroborative evidence for extensive crosslink cleavage. The SEM morphology provides indirect supporting evidence by showing the physical disruption of the rubber network. Thus, while each technique has limitations individually, the convergence of evidence across all four characterization methods—sol fraction, FTIR, EDS, and SEM—establishes a coherent picture of selective crosslink cleavage.

### 3.4. Conventional Properties of Modified Asphalts

**Figure 6:**
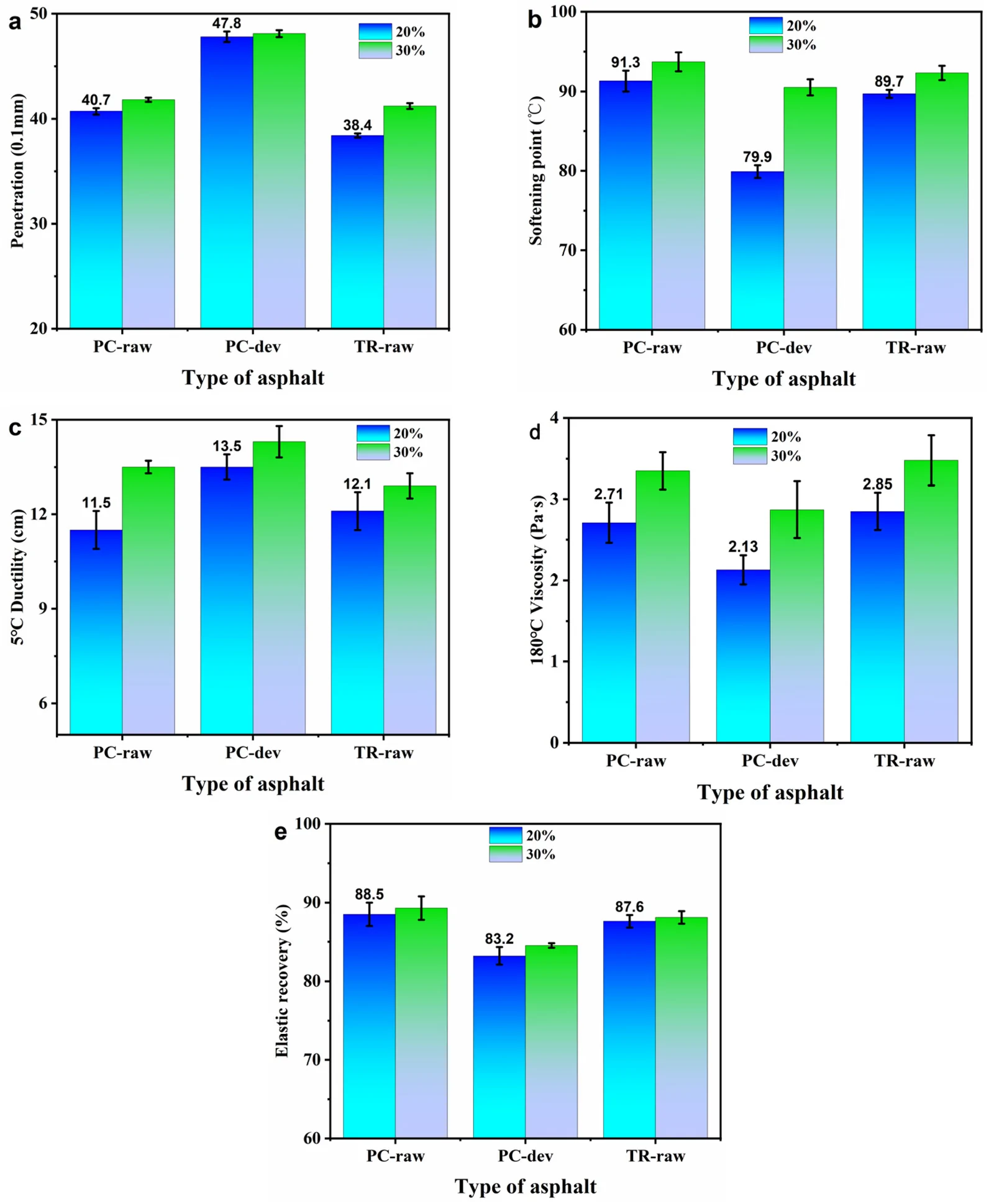
Technical indexes of modified asphalts: (**a**) penetration, (**b**) softening point, (**c**) 5 °C ductility, (**d**) 180 °C viscosity and (**e**) elastic recovery.

The conventional properties of the three modified asphalts at 20 wt% and 30 wt% rubber dosages are exhibited in Figure 6.

Comparative Analysis of PC-dev vs. PC-raw:

PC-dev-modified asphalts exhibit higher penetration (47.8–48.1 vs. 40.7–41.8), superior ductility (13.5–14.3 vs. 11.5–13.5 cm), and lower viscosity (2.13–2.87 vs. 2.71–3.35 Pa·s) compared to PC-raw. These differences are directly attributable to the microstructural changes induced by devulcanization. The increased sol fraction (29.3% vs. 8.8%) releases linear polymer chains that plasticize the asphalt, while the rough, porous morphology (SEM) enhances interfacial interaction. FTIR and EDS confirm crosslink cleavage (S=O at 1030 cm^−1^; S: 11.4 → 0.6 wt%), reducing network density and lowering stiffness. This explains the reduced softening point of PC-dev at 20 wt% dosage (79.9 vs. 91.3 °C). However, at 30 wt% dosage, PC-dev-30 reaches 90.5 °C, approaching PC-raw-30 (93.7 °C), demonstrating that higher rubber content compensates for crosslink loss. Slightly lower elastic recovery (83.2–84.5 vs. 88.5–89.3%) is also a consequence of reduced crosslink density.

Comparative Analysis of PC-dev vs. TR-raw and Dosage Effects:

PC-dev consistently outperforms TR-raw in ductility (13.5–14.3 vs. 12.1–12.9 cm) and workability (lower viscosity: 2.13–2.87 vs. 2.85–3.48 Pa·s), while TR-raw shows higher softening point (89.7–92.3 vs. 79.9–90.5 °C) and elastic recovery (87.6–88.1 vs. 83.2–84.5%). These differences arise from TR-raw’s intact crosslinked network (S: 15.5 wt%; sol fraction: ~8.8%), providing greater stiffness but poorer low-temperature flexibility and workability. Notably, increasing PC-dev dosage from 20 wt% to 30 wt% elevates softening point by 10.6 °C (79.9 → 90.5 °C) and viscosity by 34.7% (2.13 → 2.87 Pa·s), approaching TR-raw levels, while maintaining superior ductility and penetration. The 3 wt% SBS component enhances elastic recovery across all binders (>80%), compensating for devulcanization-induced crosslink loss. These results confirm that high-dosage PC-dev/SBS composite modification offers a balanced performance profile—comparable high-temperature rutting resistance to TR-raw, with superior low-temperature crack resistance and workability—making devulcanized passenger car tire rubber a viable alternative to truck tire rubber in asphalt applications.

For reference, the JTG F40–2004 specification for SBS-modified asphalt (I-D grade) requires a minimum softening point of 60 °C, a minimum 5 °C ductility of 20 cm, and a minimum elastic recovery of 75%. All PC-dev-modified asphalts meet or exceed these requirements, with PC-dev-30 achieving 90.5 °C softening point, 14.3 cm ductility, and 84.5% elastic recovery. While the ductility of PC-dev-30 (14.3 cm) is below the I-D grade requirement (20 cm), this reflects the more demanding 5 °C ductility test compared to the standard 5 °C requirement for SBS-modified asphalt; all PC-dev samples still substantially outperform unmodified asphalt.

Compared to conventional crumb rubber-modified asphalt prepared from mixed tire sources without devulcanization, which typically exhibits softening points in the range of 65–75 °C and 5 °C ductility of 8–12 cm at 20% dosage [44], PC-dev-20 achieves a higher softening point (79.9 °C) and superior ductility (13.5 cm). The primary advantage of the devulcanized approach is the improved compatibility and workability, while the main disadvantage is the additional processing cost and the reduced stiffness at lower dosages. However, the dosage compensation mechanism identified in this study—whereby increasing the rubber content to 30% restores high-temperature performance—provides a pathway to overcome this limitation.

**Figure 7:**
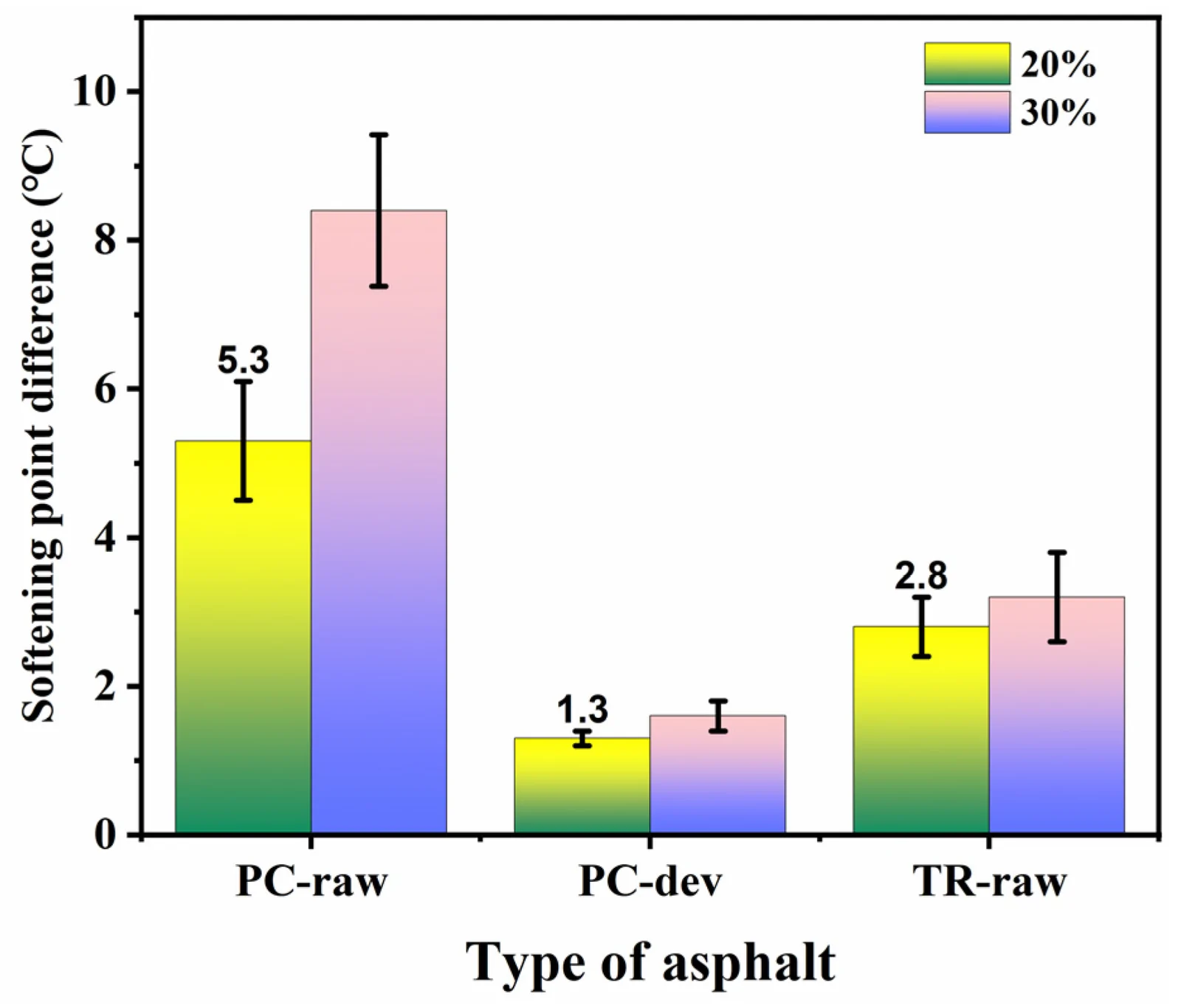
Storage stability at 163 °C of modified asphalts.

The improvement in high-temperature performance at 30% dosage is primarily attributed to the increased rubber phase volume—a dosage effect that is observed for all rubber types. However, the key role of devulcanization lies in enabling this higher dosage to be effectively utilized. As shown in the storage stability results (Figure 7), PC-raw-30 exhibits severe phase separation (ΔT = 8.4 °C), indicating that untreated PCWT rubber cannot be used at 30% dosage without compromising binder homogeneity. In contrast, PC-dev-30 maintains excellent storage stability (ΔT = 1.6 °C), demonstrating that devulcanization is essential for high-dosage application of PCWT rubber. Thus, while the stiffness improvement at 30% is largely a dosage effect, the ability to achieve this improvement without phase separation is specifically attributable to the devulcanized structure.

The storage stability results (Figure 7) show that PC-dev-modified asphalts exhibit excellent storage stability, with ΔT values of 1.3–1.6 °C, well below the acceptable limit of 2.5 °C. In contrast, PC-raw-30 exhibits severe phase separation (ΔT = 8.4 °C), confirming that untreated passenger car tire rubber is unsuitable for high-dosage applications. This further demonstrates that devulcanization effectively improves the compatibility of PCWT rubber with the asphalt/SBS matrix.

### 3.5. DSR Analysis of Modified Asphalts

**Figure 8:**
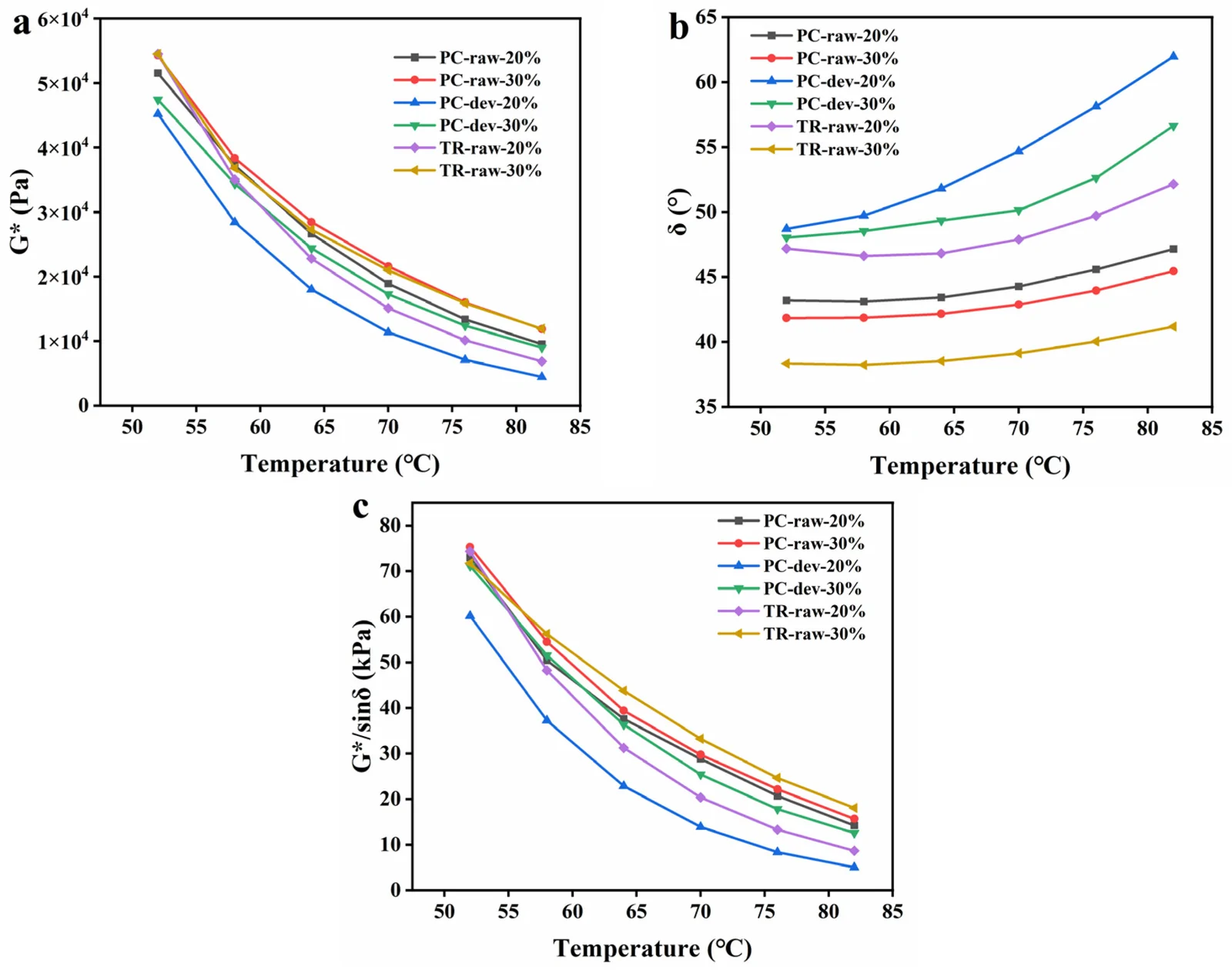
The dynamic rheological curves of modified asphalts: (**a**) complex modulus (G*), (**b**) phase angle (δ) and (**c**) rutting factor (G*/sinδ).

The DSR results, including complex modulus (G*), phase angle (δ), and rutting factor (G*/sinδ), are presented in Figure 8. Complex Modulus (G*). Complex Modulus (G*). PC-dev exhibits significantly lower G values* than PC-raw and TR-raw at equivalent temperatures and dosages. This reduction in stiffness is directly attributable to the decreased crosslink density resulting from devulcanization, as confirmed by the increased sol fraction (8.8 → 29.3%), sulfur reduction (EDS: 11.4 → 0.6 wt%), and FTIR-detected S=O (1030 cm^−1^) and C=O (1710 cm^−1^) bands. The lower G* of PC-dev is consistent with its higher penetration and lower softening point observed in conventional tests, confirming that crosslink cleavage reduces the overall rigidity of the rubber phase and, consequently, the composite binder. TR-raw exhibits the highest G* values across all temperatures (e.g., at 70 °C: ~18,000 Pa), reflecting its intact crosslinked network and higher sulfur content (EDS: 15.5 wt%). Notably, PC-raw-20 and PC-raw-30 show nearly identical G* values at each temperature, indicating that increasing the dosage of untreated rubber does not proportionally increase stiffness due to poor dispersion and limited interfacial interaction. In contrast, PC-dev shows a slight dosage-dependent increase in G* at higher temperatures, suggesting that the improved dispersion of devulcanized rubber allows the additional rubber content to contribute more effectively to the composite network.

Phase Angle (δ). PC-dev exhibits significantly higher δ values (48–52°) than PC-raw (42–47°) and TR-raw (38–43°) across all temperatures, indicating a more viscous, less elastic response. This is mechanistically consistent with the reduced crosslink density from devulcanization, which decreases the elastic contribution of the rubber phase. The higher δ of PC-dev aligns with its lower elastic recovery in conventional tests (83.2–84.5 vs. 88.5–89.3%), confirming that crosslink cleavage shifts the binder response toward viscous behavior. TR-raw shows the lowest δ, reflecting its intact vulcanized network and predominantly elastic response.

Rutting factor (G*/sinδ). Due to the relatively low G* and high δ values of PC-dev, its G*/sinδ value is significantly lower than that of PC-raw and TR-raw. However, at a 30 wt% dosage, the gap narrows further (for example, at 70 °C: PC-dev-30 ~ 16,000 Pa vs. PC-raw-30 ~ 18,000 Pa), indicating that increasing the rubber content can partially compensate for the stiffness reduction caused by sulfurization. Among them, the 3 wt% SBS component plays a key role in this compensation mechanism: The SBS network provides sufficient elastic recovery ability, enabling it to maintain good rutting resistance even when the rubber interconnection density decreases. The similarity of the G/sinδ values confirms that the PC-dev/SBS composite-modified asphalt has sufficient high-temperature performance, which is consistent with the softening point results, that is, PC-dev-30 reaches 90.5 °C, close to 93.7 °C of PC-raw-30. Due to its intact crosslinking structure, TR-raw maintains the highest G/sinδ value under all conditions.

The lower complex modulus (G) of PC-dev compared to PC-raw is primarily attributable to the reduced crosslink density resulting from devulcanization, as directly evidenced by the increased sol fraction (8.8 → 29.3%) and sulfur reduction (11.4 → 0.6 wt%). The higher phase angle (δ) of PC-dev reflects the more viscous nature of the devulcanized rubber phase, consistent with its lower elastic recovery in conventional tests. The improved low-temperature performance of PC-dev (higher m-value, lower S) is attributed to the flexible polymer chains released upon crosslink cleavage, which plasticize the asphalt matrix. Meanwhile, the 3% SBS network provides a counterbalancing elastic contribution that maintains adequate rutting resistance (G/sinδ) despite the reduced crosslink density of the rubber phase. Thus, the rheological response of PC-dev-modified asphalts reflects a combination of: (a) reduced rubber crosslink density (lower G*, higher δ), (b) released flexible polymer chains (improved low-temperature performance), and (c) the SBS network (maintained rutting resistance).

### 3.6. MSCR Analysis of Modified Asphalts

**Figure 9:**
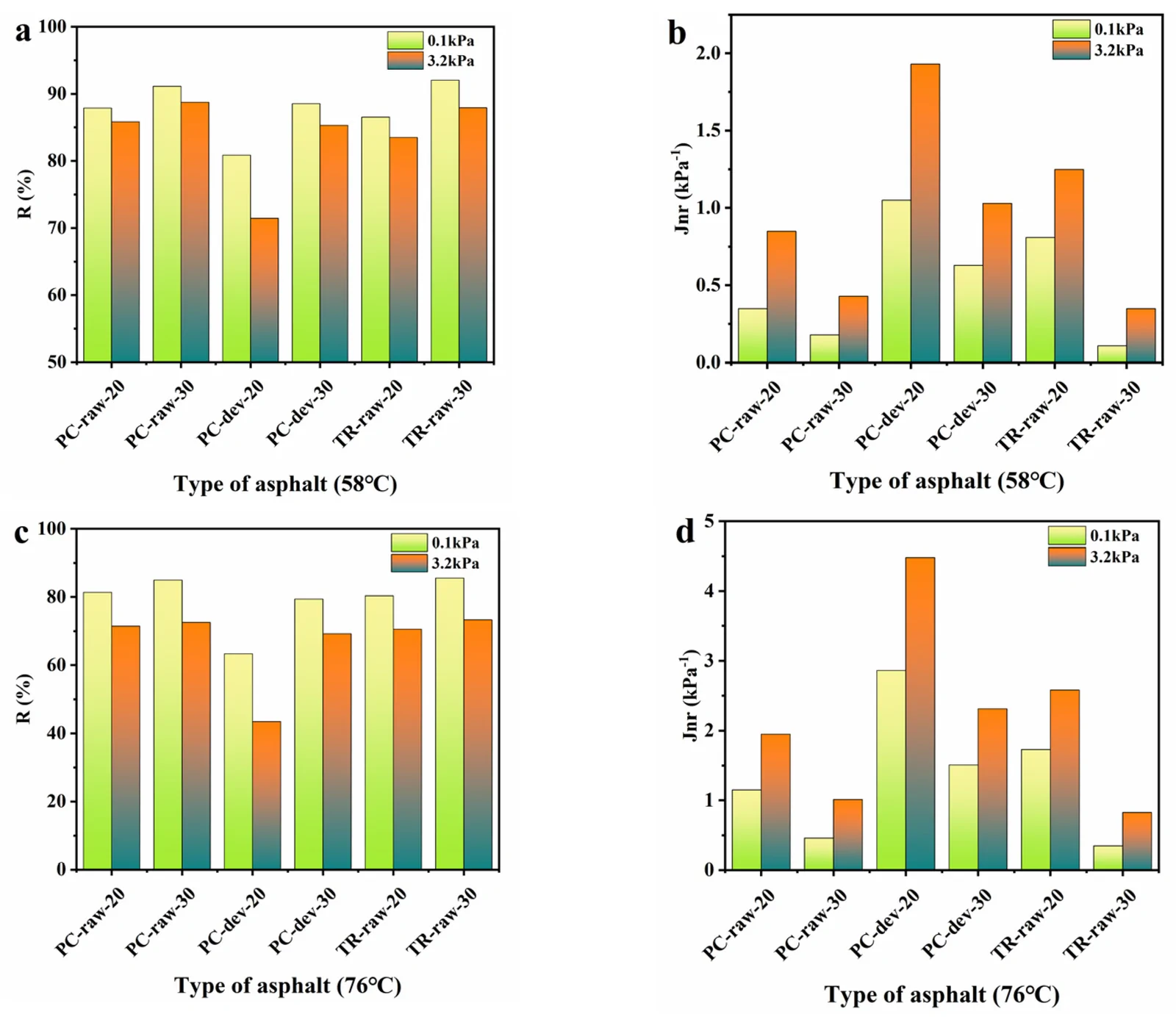
Recovery and *J_nr_* of modified asphalts: (**a**) R at 58 °C, (**b**) *J_nr_* at 58 °C, (**c**) R at 76 °C and (**d**) *J_nr_* at 76 °C.

The MSCR test results at 58 °C and 76 °C are presented in Figure 9, with percent recovery (R) and non-recoverable creep compliance (*J_nr_*) at 0.1 kPa and 3.2 kPa.

Recovery (R) and *J_nr_* at 58 °C. PC-dev-20 exhibits substantially lower R (81% at 0.1 kPa, 71% at 3.2 kPa) and significantly higher *J_nr_* (1.05–1.95 kPa^−1^) compared to PC-raw-20 (R: 88–87%; *J_nr_*: 0.35–0.88 kPa^−1^) and TR-raw-20 (R: 87–84%; *J_nr_*: 0.85–1.25 kPa^−1^). This indicates that devulcanization reduces elastic recovery and increases permanent deformation, consistent with crosslink cleavage evidenced by the increased sol fraction (8.8 → 29.3%), sulfur reduction (EDS: 11.4 → 0.6 wt%), and enhanced FTIR bands at 1030 cm^−1^ (S=O) and 1710 cm^−1^ (C=O). The higher *J_nr_* values of PC-dev directly reflect the reduced crosslink density and the more viscous response observed in DSR (higher phase angle). Conversely, PC-raw-30 and TR-raw-30 exhibit the lowest *J_nr_* (0.08–0.45 kPa^−1^) and highest R (89–92%), attributed to their intact crosslinked networks and higher sulfur content (TR-raw: 15.5 wt%), which provide greater elastic recovery.

Dosage Effect. Increasing PC-dev content from 20 wt% to 30 wt% significantly improves R (81 → 89% at 0.1 kPa, 71 → 85% at 3.2 kPa) and reduces *J_nr_* (1.05 → 0.65 kPa^−1^ at 0.1 kPa, 1.95 → 1.05 kPa^−1^ at 3.2 kPa). At 76 °C, similar trends are observed: PC-dev-30 shows higher R (79–69%) and lower *J_nr_* (1.5–2.3 kPa^−1^) than PC-dev-20 (63–43%; 2.8–4.6 kPa^−1^). This dosage-dependent improvement is attributed to the increased rubber phase volume, which enhances the SBS/rubber composite network and partially compensates for the reduced crosslink density caused by devulcanization. PC-raw-30 and TR-raw-30 consistently outperform their 20 wt% counterparts, with TR-raw-30 achieving the lowest *J_nr_* (0.08–0.35 kPa^−1^ at 58 °C), reflecting the combined benefits of high rubber content and intact crosslinked structure. The porous morphology of PC-dev (SEM) enhances interfacial bonding but cannot fully offset the intrinsic crosslink loss. Overall, PC-dev-30 achieves comparable rutting resistance to PC-raw-20 and TR-raw-20, confirming that high-dosage PC-dev/SBS composite modification is a viable strategy for balancing elasticity, workability, and deformation resistance.

### 3.7. BBR Analysis of Modified Asphalts

**Figure 10:**
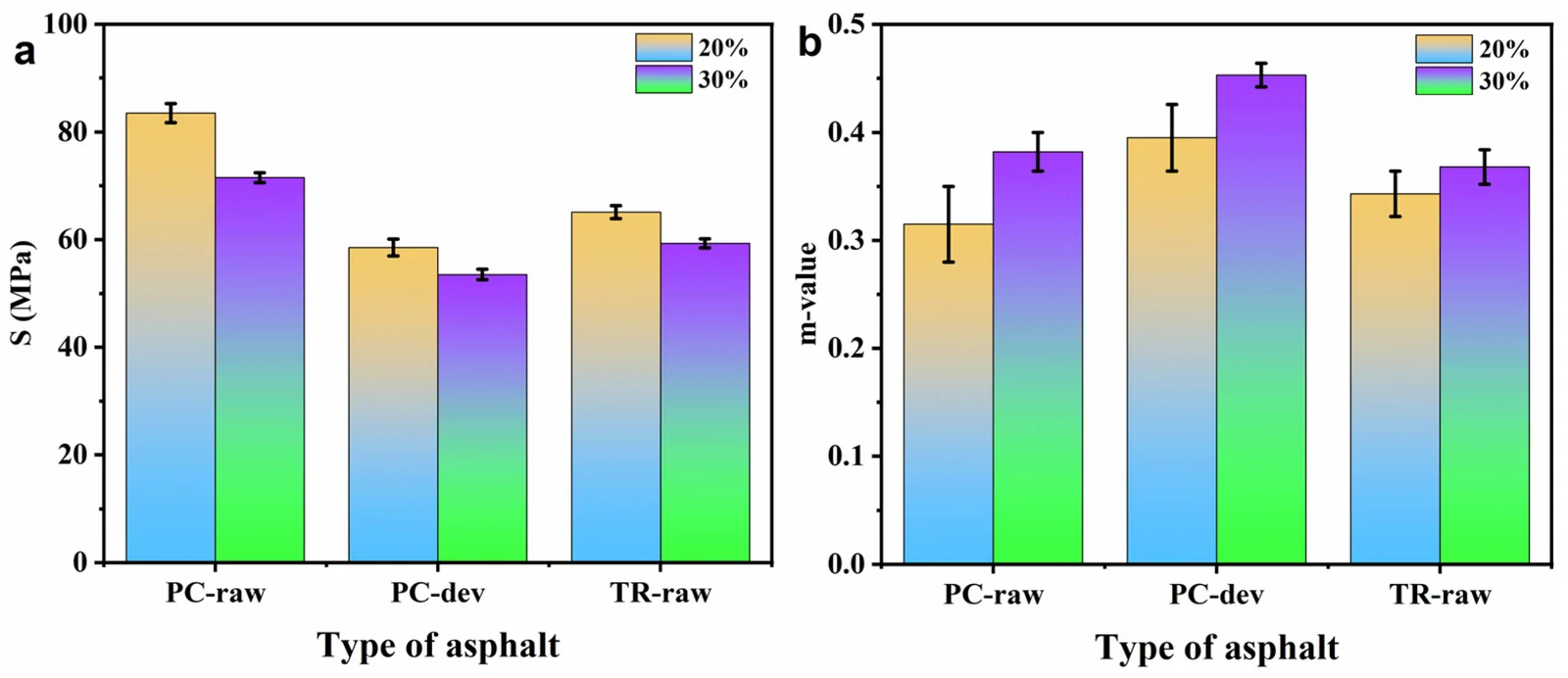
(**a**) S and (**b**) m-value of modified asphalts.

The BBR tests of the three modified asphalts are shown in Figure 10. At −18 °C. PC-dev exhibits the lowest creep stiffness (S) at both dosages (59 MPa at 20 wt%, 56 MPa at 30 wt%) compared to PC-raw (84 → 73 MPa) and TR-raw (66 → 60 MPa). Concurrently, PC-dev shows the highest m-values (0.39 → 0.46), indicating superior stress relaxation capacity. This confirms that devulcanized rubber significantly enhances low-temperature crack resistance, consistent with the superior ductility observed in conventional tests.

The improved low-temperature performance of PC-dev is attributed to the microstructural changes from devulcanization: the increased sol fraction (8.8 → 29.3%) releases flexible polymer chains that plasticize the asphalt matrix, while the rough, porous morphology (SEM) enhances interfacial bonding. FTIR and EDS confirm crosslink cleavage (S: 11.4 → 0.6 wt%), reducing network rigidity and enabling better molecular chain mobility at low temperatures. The higher m-values indicate faster stress relaxation, crucial for preventing thermal cracking. Increasing dosage from 20 wt% to 30 wt% further improves performance for all binders, as the higher rubber content provides more flexible polymer segments. PC-dev-30 achieves the best combination of low stiffness and high relaxation ability, consistent with its higher ductility and workability observed in conventional and rheological tests.

**Figure 11:**
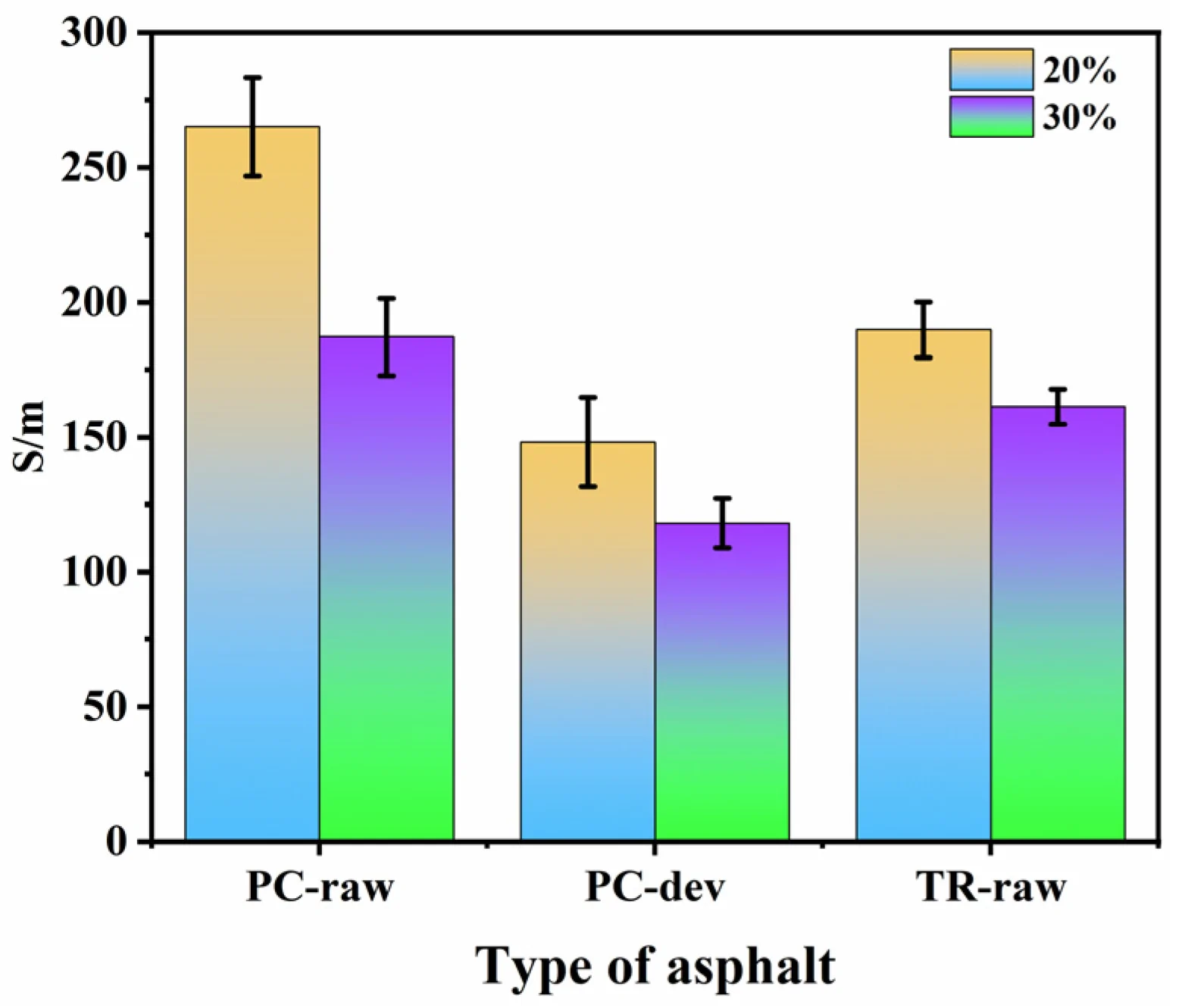
S/m of modified asphalts.

The S/m value is also used to analyze the low temperature performance of modified asphalt in related reports [45]. As shown in Figure 11, PC-dev-30 exhibits the lowest S/m value (~250), indicating the best low-temperature cracking resistance. A lower S/m value signifies superior stress relaxation capability at reduced stiffness levels, consistent with its highest 5 °C ductility (14.3 cm) in conventional tests. This improvement is attributed to the flexible molecular chains released upon devulcanization, which toughen the asphalt matrix. TR-raw-30 and PC-raw-30 show higher S/m values (~432 and ~487), suggesting relatively poorer low-temperature performance due to their intact crosslinked networks that restrict molecular mobility.

It is important to recognize that PCWT and truck tire rubber differ not only in crosslink density but also in intrinsic rubber composition: PCWT contains predominantly SBR (~65%), while TR predominantly contains NR (~65%) [46]. The higher SBR content of PCWT contributes to its better low-temperature flexibility after devulcanization, as SBR has a lower glass transition temperature than NR. Conversely, the higher NR content of TR contributes to its greater stiffness and elastic recovery, as NR has a higher tendency to crystallize upon stretching. Thus, the observed differences between PC-dev and TR-raw reflect a combination of: (a) the effect of devulcanization (reduced crosslink density in PC-dev), (b) the intrinsic compositional differences between SBR-rich and NR-rich rubbers, and (c) the interaction of both with the SBS/asphalt matrix. While devulcanization is the primary factor enabling PCWT rubber to achieve performance comparable to TR, the compositional differences also play a role and should be considered in the interpretation of the results.

## 4. Conclusions

This study systematically evaluated the effects of HDA-assisted thermo-mechanical devulcanization on passenger car tire rubber and its subsequent performance in SBS-composite-modified asphalt at 20 wt% and 30 wt% rubber dosages. The following conclusions are drawn:

(1) HDA-assisted thermo-mechanical devulcanization selectively cleaves C–S and S–S crosslinks while preserving the rubber backbone. Direct evidence includes the increased sol fraction (8.8 → 29.3%) and preserved C-H stretching (2911/2843 cm^−1^) in FTIR. Supporting evidence includes enhanced S=O (1030 cm^−1^) and C=O (1710 cm^−1^) FTIR bands, sulfur reduction (11.4 → 0.6 wt%), and rough porous morphology (SEM). These changes fundamentally improve the compatibility of PCWT rubber with asphalt.

(2) PC-dev-modified asphalts exhibit a dosage-dependent performance trade-off: at 20 wt% dosage, they show superior low-temperature flexibility and workability but reduced high-temperature stiffness compared to PC-raw and TR-raw. Increasing the dosage to 30 wt% effectively compensates for the stiffness loss, with PC-dev-30 achieving a softening point of 90.5 °C, approaching PC-raw-30 (93.7 °C) and TR-raw-30 (92.3 °C), while maintaining superior ductility and lower viscosity.

(3) The 3 wt% SBS network governs the overall rheological response. At 20 wt% dosage, PC-dev-20 shows lower G/sinδ than PC-raw-20 and TR-raw-20 due to reduced crosslink density. However, at 30 wt% dosage, the gap narrows substantially, with PC-dev-30 achieving G/sinδ values approaching those of PC-raw-30 and TR-raw-30 (e.g., at 70 °C: ~16,000 vs. ~18,000 Pa). This confirms that a high rubber dosage compensates for the reduced crosslink density.

Application Value: HDA-assisted devulcanized passenger car tire rubber at a 30 wt% dosage with 3 wt% SBS offers a balanced performance profile comparable to truck tire rubber, with enhanced low-temperature crack resistance and workability. This provides a practical pathway for high-value utilization of passenger car waste tires in asphalt pavements, contributing to sustainable waste management and circular economy objectives.

The scientific novelty of this work lies in the first systematic demonstration that HDA-assisted thermo-mechanical devulcanization, combined with a dosage compensation strategy, can transform passenger car tire rubber—traditionally considered unsuitable for asphalt modification—into a modifier with performance comparable to truck tire rubber. Limitations of the proposed approach include the cost of HDA (approximately USD 16/kg), the energy intensity of the twin-screw extrusion process, and the need for further scale-up validation. Future work should address these economic and engineering challenges to facilitate industrial implementation.

We acknowledge that long-term thermal and photo-oxidative stability of the PC-dev-modified asphalts was not investigated in this study. While the 3% SBS component is known to impart some resistance to thermal and UV aging, quantitative aging data—such as RTFOT/PAV aging indices or UV aging resistance—were beyond the scope of this work. Future studies should systematically evaluate the aging resistance of PC-dev/SBS composite-modified asphalts to fully assess their service life in pavement applications.

## Figures and Tables

**Table 1:** Basic properties of 90# matrix asphalt.

Serial Number	Subject	Unit	Rated Value	Measured Value
1	Penetration (25 °C)	0.1 mm	80~100	88
2	Penetration index	/	−1.0~+1.0	−0.6
3	Ductility (10 °C)	cm	≥25	>100
	Ductility (15 °C)	cm	≥100	>100
4	Softening point	°C	≥45	46.5
5	Density	g/cm^3^	≥1.01	1.035
6	Solubility	%	≥99.5	99.88
7	Flash point	°C	≥245	291
8	Mass change	%	±0.8	0.062
9	Residual penetration ratio (25 °C)	%	≥57	65.4
10	Residual ductility (10 °C)	cm	≥8	11

**Table 2:** Technical index of SBS modifier.

**Structure Type**	**Block Ratio (S/B)**	**Oil Content** **%**	**Volatile Matter** **%**	**Ash Content** **%**
Linear	30/70	0	≤0.7	≤0.2
**Stress at 300% Elongation** **MPa**	**Tensile Strength** **MPa**	**Elongation at Break** **%**	**Permanent Set at Break** **%**	**Melt Flow Rate (MFR)** **G10^−1^ min^−1^**
≥2.2	≥16.0	≥700	≤40	0.5~2.5

**Table 3:** Preparation parameters for PC-dev.

Sample	Temperatures (°C)	Rotational Speeds (rpm)	HDA Contents (wt%)
PC-dev-T140R80H5	140	80	5
PC-dev-T160R80H5	160	80	5
PC-dev-T180R80H5	180	80	5
PC-dev-T160R40H5	160	40	5
PC-dev-T160R60H5	160	60	5
PC-dev-T160R80H10	160	80	10
PC-dev-T160R80H15	160	80	15

**Table 4:** Table of component content of modified asphalt.

Sample ID	Base Asphalt (wt%)	SBS Content (wt%, External Addition)	Crumb Rubber Type	Crumb Rubber Content (wt%, External Addition)
PC-raw-20	100	3	PC-raw	20
PC-raw-30	100	3	PC-raw	30
PC-dev-20	100	3	PC-dev	20
PC-dev-30	100	3	PC-dev	30
TR-raw-20	100	3	TR-raw	20
TR-raw-30	100	3	TR-raw	30

**Table 5:** The elemental contents of PC-raw, PC-dev, and TR-raw.

Sample	C (wt%)	O (wt%)	S (wt%)	N (wt%)
PC-raw	77.3	10.3	11.4	0.9
PC-dev	79.9	20.1	0.6	0.3
TR-raw	74.4	7.4	15.5	2.7

## Data Availability

The original contributions presented in this study are included in the article. Further inquiries can be directed to the corresponding author.

## Abbreviations

The following abbreviations are used in this manuscript:

PCWT	Passenger Car Waste Tire
PC-raw	Untreated Passenger Car Tire Rubber
PC-dev	Devulcanized Passenger Car Tire Rubber
TR-raw	Untreated Truck Tire Radial Rubber
HDA	Hexadecylamine
CRMA	Crumb Rubber-Modified Asphalt
SBR	Styrene-Butadiene Rubber
SBS	Styrene-Butadiene-Styrene

## References

[1] Cui D., Bi Z.H., Wang Y., Gu Y.L., Wang H.M., Gao X.F., Wang P., Sun X., Chen W.Q. (2024). Scenario Analysis of Waste Tires from China’s Vehicles Future. J. Clean. Prod..

[2] Chen B., Zheng D., Xu R., Leng S., Han L., Zhang Q., Liu N., Dai C., Wu B., Yu G. (2022). Disposal Methods for Used Passenger Car Tires: One of the Fastest Growing Solid Wastes in China. Green Energy Environ..

[3] Hamidinasab B., Nabavi-Pelesaraei A. (2025). Systematic Review on Environmental Impact Assessment of Incineration Technologies. Energy Convers. Manag. X.

[4] Boré A., Cui J., Chen G., Themelis N.J., Ma W. (2025). Understanding the Evolution of Municipal Solid Waste Incineration Emissions in China: Patterns, Determinants, and Control Measures. Atmos. Pollut. Res..

[5] Vaverková M.D. (2019). Landfill Impacts on the Environment—Review. Geosciences.

[6] Zhu J., Birgisson B., Kringos N. (2014). Polymer Modification of Bitumen: Advances and Challenges. Eur. Polym. J..

[7] Yang Q.L., Lin J., Wang X.W., Wang D.W., Xie N., Shi X.M. (2024). A Review of Polymer-Modified Asphalt Binder: Modification Mechanisms and Mechanical Properties. Clean. Mater..

[8] Cui Y., Zhang L., Wang H., Xing C., Tan Y. (2026). From Waste to Performance Evolution: Unveiling a Critical Aging Stage in Crumb Rubber Modified Asphalt for Sustainable Pavement Engineering. Constr. Build. Mater..

[9] Boateng K.A., Wu M., Jin D., You Z. (2026). Recycling Aged Crumb Rubber-Modified Asphalt Pavements: A Review. J. Road Eng..

[10] Kokate L.K., Damgir R.M. (2024). From Waste to Resource: An Interdisciplinary Analysis of Crumb Rubber Modified Asphalt for Sustainable Pavement Solutions. World J. Adv. Eng. Technol. Sci..

[11] Bisschop R., Grunert F., Ilisch S., Stratton T., Blume A. (2021). Influence of Molecular Properties of Ssbr and Br Types on Composite Performance. Polym. Test..

[12] Shi R.F., Ma J., Song X.Y., Zhan B.C., Xu X.F., Zhao S.L., He J.H. (2023). Composition Effects on Thermodynamic Properties and Interfacial Structure in Styrene-Butadiene Rubber: A Combined Experimental and Simulation Study. Chem. Eng. Sci..

[13] Marzocca A.J., Mansilla M.A., Beccar Varela M.P., Mariani M.C. (2025). Understanding the Dynamic Loss Modulus of Nr/Sbr Blends in the Glassy–Rubbery Transition Zone. Polymers.

[14] Klat D., Karimi-Varzaneh H.A., Lacayo-Pineda J. (2018). Phase Morphology of Nr/Sbr Blends: Effect of Curing Temperature and Curing Time. Polymers.

[15] Pyshye S., Lypko Y., Demchuk Y., Kukhar O., Korchak B., Pochapska I., Zhytnetskyi I. (2024). Characteristics and Applications of Waste Tire Pyrolysis Products: A Review. Chem. Chem. Technol..

[16] Seghar S., Asaro L., Rolland-Monnet M., Aït Hocine N. (2019). Thermo-Mechanical Devulcanization and Recycling of Rubber Industry Waste. Resour. Conserv. Recycl..

[17] Dorigato A., Rigotti D., Fredi G. (2023). Recent Advances in the Devulcanization Technologies of Industrially Relevant Sulfur-Vulcanized Elastomers. Adv. Ind. Eng. Polym. Res..

[18] Mohammadi M., Ishak M.R., Sultan M.T.H. (2024). Exploring Chemical and Physical Advancements in Surface Modification Techniques of Natural Fiber Reinforced Composite: A Comprehensive Review. J. Nat. Fibers.

[19] Ebrahimi M. (2026). Innovative Physical and Chemical Strategies for the Modification and Development of Polymeric Microfiltration Membranes—A Review. Polymers.

[20] Simon D.Á., Bárány T. (2021). Effective Thermomechanical Devulcanization of Ground Tire Rubber with a Co-Rotating Twin-Screw Extruder. Polym. Degrad. Stab..

[21] Costamagna M., Brunella V., Luda M.P., Romagnolli U., Muscato B., Girotto M., Baricco M., Rizzi P. (2022). Environmental Assessment of Rubber Recycling through an Innovative Thermo-Mechanical Devulcanization Process Using a Co-Rotating Twin-Screw Extruder. J. Clean. Prod..

[22] Parsamanesh M., Abbassi-Sourki F., Karrabi M., Soltani S. (2024). Thermomechanical Devulcanization of Butyl Rubber Using Twin-Screw Extruder: Process Parameters, Viscoelastic and Compatibility Properties. Prog. Rubber Plast. Recycl. Technol..

[23] Vahdatbin M., Jamshidi M. (2022). Using Chemical Agent in Microwave Assisted Devulcanization of Nr/Sbr Blends: An Effective Recycling Method. Resour. Conserv. Recycl..

[24] Guo L., Bai L.C., Zhao J.Y., Liu K.X., Jian X.G., Chai H.L., Liu F.M., Guo S.Y., Liu G.X., Liu H.C. (2024). Enhancing Devulcanizing Degree and Efficiency of Reclaimed Rubber by Using Alcoholic Amines as the Devulcanizing Agent in Low-Temperature Mechano–Chemical Process. Polymers.

[25] Vahdatbin M., Hajikarimi P., Fini E.H. (2025). Devulcanization of Waste Tire Rubber via Microwave and Biological Methods: A Review. Polymers.

[26] Kurup V.R., Sabarinath S., Shankar B., Balachandran M., Varma R.S., Padil V.V.T., Kurien R.A., Raj V., Rajeev S.P. (2026). Effect of Cross-Linking on the Mechanical, Thermal, and Tribological Behavior of the Epoxy Variants Using Response Surface Methodology. Results Eng..

[27] Wiśniewska P., Wang S., Formela K. (2022). Waste Tire Rubber Devulcanization Technologies: State-of-the-Art, Limitations and Future Perspectives. Waste Manag..

[28] Huang S., Kong X., Xiong Y.S., Zhang X.R., Chen H., Jiang W.Q., Niu Y.Z., Xu W.L., Ren C.G. (2020). An Overview of Dynamic Covalent Bonds in Polymer Material and Their Applications. Eur. Polym. J..

[29] Guo F.Q., Shen Z.L., Yu Z.W., Jiang L.Z., Long Q.L., He C. (2024). Performance Study of Sbs/Crma with Different Composite Crumb Rubber Particle Size Ratios. Case Stud. Constr. Mater..

[30] Shi C.G., Zhou W.X., Wang T.L., Cai X., Wang H.Z., Yang J., Hong J.X., Gong M.H. (2022). Fatigue Performance Characterization of Styrene-Butadiene-Styrene and Crumb Rubber Composite Modified Asphalt Binders with High Crumb Rubber Contents. J. Clean. Prod..

[31] Jia X.F., Li Q.D., Zhao X., Han S.B. (2025). Effect of Crumb Rubber Activation on the Rheological and Aging Properties of Crumb Rubber/Sbs Modified Asphalt. Int. J. Pavement Res. Technol..

[32] Chen X.W., Zhang L.N., Liu J.J., Xi X.Y., Yu R.E., Cheng X. (2025). Preparation and Properties of Synergistic Activated Rubber Powder/Sbs Composite Modified Asphalt for Waterproof Coatings. Case Stud. Constr. Mater..

[33] Guo M., Guo Y.F., Sun G.Q., Yang C., Luo D.S. (2025). Preparation and Evaluation of Compatibilized Sbs/Cr Composite-Modified Asphalt by Exploiting Ccd-Rsm: A Higher Value Utilization Pathway for Waste Rubber. Constr. Build. Mater..

[34] Zhou S.X., Yan J.Q., Li L.Y., Li S.Q., Wu S.M., Yan C.Q., Ai C.F. (2024). Investigating the Influence of Stress Dependency on the Evaluation of High-Temperature Deformation Resistance in Sbs-Modified Asphalt. Constr. Build. Mater..

[35] Meng Y.Y., Chen P., Pang Y.F., Huang K., Wan C., Shen T. (2026). Performance, Modification Mechanism and Cost-Benefit Analysis of Sbs/Terminal Blended Rubberized Composite High Viscosity Modified Asphalt. Constr. Build. Mater..

[36] Veselý P., Dašek O., Jasso M. (2026). Optimizing Asphalt Modifications: Interactions between Sbs and Ppa Modifiers. Infrastructures.

[37] Wu M., Boateng K.A., Yin L., Liu Z., You Z., Jin D. (2025). High-Content Crumb Rubber Modified Asphalt Mixture via Wet Process: Laboratory Evaluation and Field Application. Constr. Build. Mater..

[38] Kang B.D., Li G.Y., Qi C., Zhang H.G., Han S.J., Kuang D.L., Chen H.X., Wu Y.C. (2025). Influence of Crumb Rubber Mechanochemical De-Crosslinking on the Microstructure, Rheological Behavior, and Aging Resistance of Modified Asphalt. Constr. Build. Mater..

[39] Rajawasam C., Dodo O., Weerasinghe M., Raji I., Wanasinghe S., Konkolewicz D., Watuthanthrige N.D.A. (2024). Educational Series: Characterizing Crosslinked Polymer Networks. Polym. Chem..

[40] Ol’khov Y.A., Lodygina V., Baturin S., Kuzayev A. (1985). Properties of Sol Fractions in Crosslinked Polyether Urethanes. Polym. Sci. USSR.

[41] Valdés C., Guzmán V., Mamani M., Vergara C., Andler R. (2025). Desulfurization of Vulcanized Rubber by a Synergism of Chemical and Biological Treatment, with the Strains Gordonia Desulfuricans and Rhodococcus Erythropolis. Polym. Test..

[42] Xu Z.P., Lin M., Jiang W.B., Xu L.G., Ji L.L., Cao G.H., Hao W. (2023). Pore-Morphology-Based Pore Structure Characterization for Various Porous Media. Chem. Eng. Sci..

[43] Kong Y., Chen X., Li Z., Li G., Huang Y. (2023). Evolution of Crosslinking Structure in Vulcanized Natural Rubber During Thermal Aging in the Presence of a Constant Compressive Stress. Polym. Degrad. Stab..

[44] Nagurskyy A., Grynyshyn O., Khlibyshyn Y., Korchak B. (2023). Use of Rubber Crumb Obtained from Waste Car Tires for the Production of Road Bitumen and Roofing Materials from Residues of Ukrainian Oil Processing. Chem. Chem. Technol..

[45] Liu S.T., Cao W.D., Shang S.J., Qi H., Fang J.G. (2010). Analysis and Application of Relationships between Low-Temperature Rheological Performance Parameters of Asphalt Binders. Constr. Build. Mater..

[46] Ahmed K.Z., Faizan M. (2024). Comprehensive Characterization of Waste Tire Rubber Powder. J. Inst. Eng. (India) Ser. E.

